# Azilsartan improves doxorubicin-induced cardiotoxicity via inhibiting oxidative stress, proinflammatory pathway, and apoptosis

**DOI:** 10.25122/jml-2023-0106

**Published:** 2023-12

**Authors:** Mohammed Al-Chlaihawi, Ali Janabi

**Affiliations:** 1 Department of Pharmacy, Kufa Technical Institute, Al-Furat Al-Awsat Technical University, Najaf, Iraq; 2 Department of Pharmacology and Toxicology, Faculty of Pharmacy, University of Kufa, Najaf, Iraq

**Keywords:** Azilsartan, inflammatory markers, doxorubicin-induced cardiomyopathy, oxidative stress, apoptotic factors

## Abstract

Azilsartan, a known angiotensin receptor blocker, has shown potential in reducing 24-hour blood pressure and may have protective effects against cardiac complications. Increased oxidative stress in cardiac tissue is directly related to the cardiac complications of doxorubicin. This study investigated whether azilsartan could mitigate doxorubicin-induced cardiotoxicity. We divided 28 male rats into four groups: the control group receiving a standard diet and water, the vehicle group given DMSO orally for two weeks, doxorubicin group receiving 2.5 mg/kg of doxorubicin three times a week for two weeks, and azilsartan group treated with 5 mg/kg/day of azilsartan orally and doxorubicin. Doxorubicin-induced cardiotoxicity was evidenced by a significant increase in TNF-α, IL-1β, MDA, and caspase-3 levels and significantly decreased TAC and Bcl-2 levels in the cardiac tissues of treated rats compared to the DMSO and control groups. Azilsartan significantly decreased doxorubicin-induced cardiotoxicity, as evidenced by a decline in serum levels of both TNF-α and IL-1β. Additionally, MDA significantly decreased in the cardiac tissue, although TAC was significantly increased when comparing the azilsartan group to the group receiving doxorubicin-only. These results suggest that azilsartan effectively reduced doxorubicin-induced cardiotoxicity, likely by mitigating apoptosis, inflammation, and oxidative stress in cardiac tissues.

## INTRODUCTION

Cardiotoxicity is a medical condition characterized by damage to the heart's electrical or muscular systems. This damage leads to a weakened heart, diminishing its ability to pump blood effectively. Radiation therapy, chemotherapy agents like anthracycline, anorexia nervosa issues, heavy metal exposure, chronic cocaine usage, or incorrectly administered drugs like doxorubicin (DOX) can all cause cardiotoxicity as a side effect [1]. Manifestations of cardiotoxicity vary widely, from minor blood pressure fluctuations to severe outcomes like arrhythmias and cardiomyopathy, potentially leading to death or chronic heart failure [2]. These potentially dangerous side effects could limit anthracycline use in clinical practice, affecting the quality of life and survival of cancer patients independent of their prognosis [3].

Approximately nine percent of patients undergoing a year-long chemotherapy regimen with anthracyclines experience a decline in left ventricular ejection fraction. The primary mechanisms underlying DOX-induced cardiomyopathy include oxidative stress and apoptosis-mediated loss of cardiomyocytes. A critical early event in DOX-induced cardiovascular disease is the decline in Ca2+ within the sarcoplasmic reticulum. Ca2+ and calmodulin-dependent kinase II levels have a direct relationship with heart cell apoptosis and heart failure [4]. Moreover, it is believed that mitochondrial biogenesis impairment plays a significant role in DOX-mediated cardiotoxicity, primarily through mechanisms involving topoisomerase II inhibition and cell death pathways [5].

Furthermore, DOX-induced cardiotoxicity is also associated with various other mechanisms. These include mitochondrial dysfunction, alterations in iron-regulating proteins, production of nitric oxide, inflammatory mediators, calcium equilibrium imbalance, autophagy, and cell death. A critical factor in this process is the generation of reactive oxygen species (ROS) [6]. DOX, a secondary metabolite of *Streptomyces peucetius* var. Caesius is known for its anticancer action, primarily mediated through the inhibition of Topoisomerase II and DNA complexation in rapidly dividing tumor cells. However, its therapeutic use is limited because it causes cumulative and dose-dependent cardiotoxicity, raising the mortality risk for cancer patients [6].

In this context, azilsartan, a medication used to manage hypertension, emerges as a relevant therapeutic agent. Lowering blood pressure can reduce the risk of cardiac events, strokes, and kidney problems. Azilsartan, part of the angiotensin receptor blockers (ARBs) family, works by relaxing blood vessels, thus facilitating easier blood flow [7]. The presence of angiotensin II type 1 receptor (AT1R) blockers in azilsartan elevates angiotensin II (ANG-II) levels, enhancing the activation of angiotensin II type 2 receptor (AT2R). Similarly, when AT1R blockers are present, the angiotensin-converting enzyme homolog effectively hydrolyzes ANG-II to create ANG 1-7, which activates endothelial cells to produce nitric oxide via the Mas receptor (Mas R). AT2R and Mas R activation influences the vasodilating, anti-inflammatory, and anti-proliferative effects of ARBs in cardiovascular diseases [8].

Oxidative stress, a significant pathogenic factor for cardiac hypertrophy, is induced by ANG-II binding to the AT1 receptor. This binding triggers the activation of the p38 mitogen-activated protein kinase (MAPK) and nuclear factor kappa-light-chain-enhancer of activated B cells (NF-κB)-mediated signaling pathways. Inhibiting ANG-II-induced cardiac hypertrophy is another method for successfully upregulating endogenous antioxidant systems and limiting ROS formation [9]. Azilsartan also influences the apoptotic action of DOX. While it directly activates effector caspases in some cell types, such as caspase-3, in most tumor cells, it enhances cell death signaling by activating the intrinsic apoptosis pathway through caspase-8. In response to an apoptotic stimulus, the latter is controlled by both pro-apoptotic and anti-apoptotic Bcl-2 family proteins, with variations in intrafamily protein interactions at the mitochondrial surface influencing the release of cytochrome c [10]. Studies have shown that in breast cancer cell lines, azilsartan significantly increases the expression of cleaved caspase-3, c-PARP proteins, and Bax, a pro-apoptotic factor while decreasing the expression of Bcl-2, an anti-apoptotic factor [11]. The primary aim of this study was to investigate the potential cardioprotective effects of azilsartan, an angiotensin receptor blocker, against DOX-induced cardiotoxicity.

## MATERIAL AND METHODS

### Study design and location

Twenty-eight male Sprague Dawley rats, aged 10-12 weeks and weighing 150-240 g, were provided by the Faculty of Science, University of Kufa. The animals were housed under controlled environmental conditions (24 ± 2°C, regulated humidity) in group caging systems at the animal facility at the College of Science. Access to water and standard chow was provided ad libitum, and a two-week acclimatization period allowed the animals to adapt to the new environment and mitigate relocation stress. Rats were randomly assigned into four groups (n=7 per group) at three months of age. The control group received a standard diet and water throughout the study. The dimethyl sulfoxide (DMSO) group was administered orally 10 ml/kg/day of DMSO for two weeks. The DOX group received 2.5 mg/kg of DOX intraperitoneally three times weekly for two weeks (total of 15 mg/kg) [12]. Azilsartan was administered orally at a dosage of 5 mg/kg per day for two weeks alongside DOX, which was administered intraperitoneally at a dose of 2.5 mg/kg, three times a week for two weeks, totaling a cumulative dose of 15 mg/kg [13].

### Serum and tissue homogenate preparation

The body weight of each rat was measured forty-eight hours after the last chemotherapy dose. Anesthesia was induced with 100 mg/kg ketamine and 10 mg/kg xylazine administered intravenously. Blood was collected from the left ventricle post-anesthesia via heart puncture following an abdominal incision. The collected blood was placed in tubes with thrombus formation gel and centrifuged at 4,000 rpm for 10 minutes to separate the serum. The serum was used for ELISA assays of interleukin-1 beta (IL-1β) and tumor necrosis factor-alpha (TNF-α) using commercially available kits [14]. The basal part of the heart was rinsed with ice-cold saline, homogenized in a 1:10 (w/v) solution of 0.1 M phosphate-buffered saline (pH 7.4) using an ultrasonic processor, and stored at -80°C. The supernatants were obtained, standardized, and centrifuged at 14,000 rpm for 10 minutes at four degrees Celsius to measure caspase-3, malondialdehyde (MDA), total antioxidant capacity (TAC), and B-cell lymphoma 2 (Bcl-2) according to the manufacturer’s instructions (PARSBiochem).

### Tissue sampling for histopathology

The apical portion of the heart was fixed in 10% neutral buffered formalin, embedded in paraffin, and sectioned at a thickness of 5 µm using a microtome. The tissue sections were stained with hematoxylin and eosin (H&E) and examined under light microscopy for histopathological analysis [15].

### Quantitative analysis of cardiac biomarkers

The levels of IL-1β, TNF-α, caspase-3, Bcl-2, MDA, and TAC in the serum and tissue homogenates were quantified using enzyme-linked immunosorbent assay (ELISA) kits according to the manufacturer's instructions.

### Statistical Analysis

Data were analyzed using GraphPad Prism 8 software. Results were expressed as mean ± standard error of the mean (SEM). Group comparisons were made using one-way ANOVA and post hoc analysis with the Bonferroni multiple comparison test. A p-value of <0.05 was considered statistically significant.

## RESULTS

**Figure 1:**
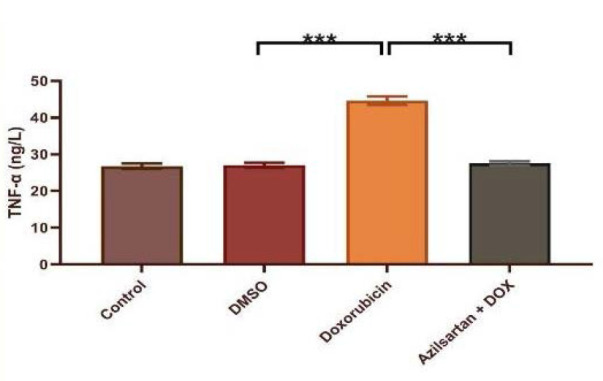
Effect of azilsartan on serum TNF-α levels Rats were administered DMSO (vehicle), DOX (2.5 mg/kg), DOX (2.5 mg/kg) plus azilsartan (5 mg/kg), or were left untreated (control). Data are expressed as mean±SEM. Statistical analysis was performed using one-way ANOVA followed by Bonferroni's multiple comparison test (***p<0.001).

**Figure 2:**
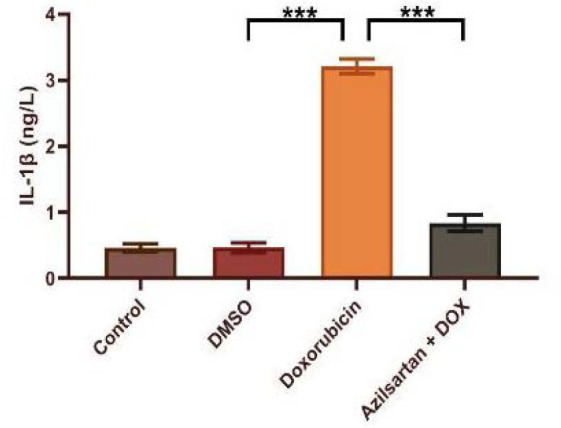
Effect of azilsartan on serum IL-1β levels Rats were administered DMSO (vehicle), DOX (2.5 mg/kg), DOX (2.5 mg/kg) plus azilsartan (5 mg/kg), or were left untreated (control). Data are expressed as mean±SEM. Statistical analysis was performed using one-way ANOVA followed by Bonferroni's multiple comparison test (***p<0.001).

**Figure 3:**
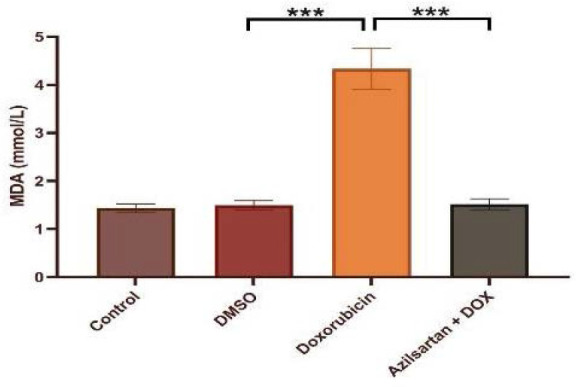
Effect of azilsartan on MDA levels Rats were administered DMSO (vehicle), DOX (2.5 mg/kg), DOX (2.5 mg/kg) plus azilsartan (5 mg/kg), or were left untreated (control). Data are expressed as mean±SEM. Statistical analysis was performed using one-way ANOVA followed by Bonferroni's multiple comparison test (***p<0.001).

**Figure 4:**
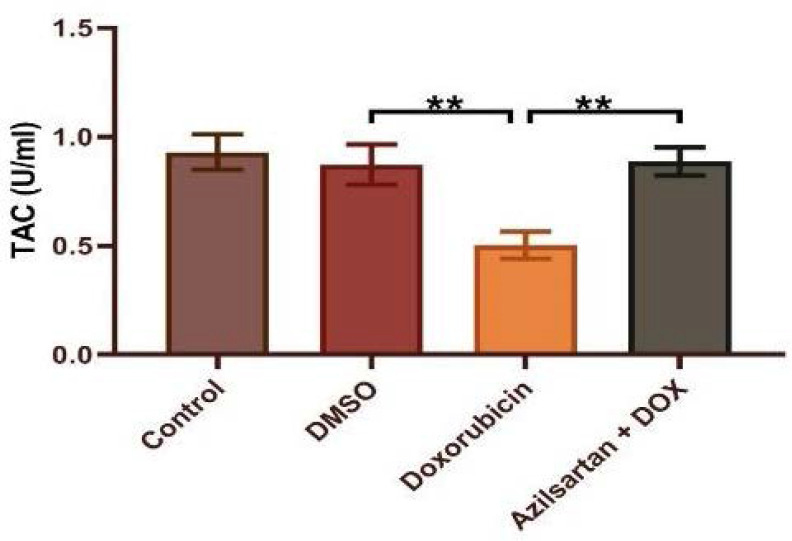
Effect of azilsartan on TAC levels Rats were administered DMSO (vehicle), DOX (2.5 mg/kg), DOX (2.5 mg/kg) plus azilsartan (5 mg/kg), or were left untreated (control). Data are expressed as mean±SEM. Statistical analysis was performed using one-way ANOVA followed by Bonferroni's multiple comparison test (**p<0.01).

**Figure 5:**
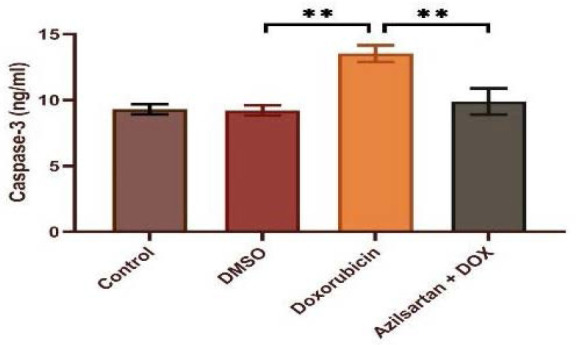
Effect of azilsartan on caspase-3 levels Rats were administered DMSO (vehicle), DOX (2.5 mg/kg), DOX (2.5 mg/kg) plus azilsartan (5 mg/kg), or were left untreated (control). Data are expressed as mean±SEM. Statistical analysis was performed using one-way ANOVA followed by Bonferroni's multiple comparison test (**p<0.01).

**Figure 6:**
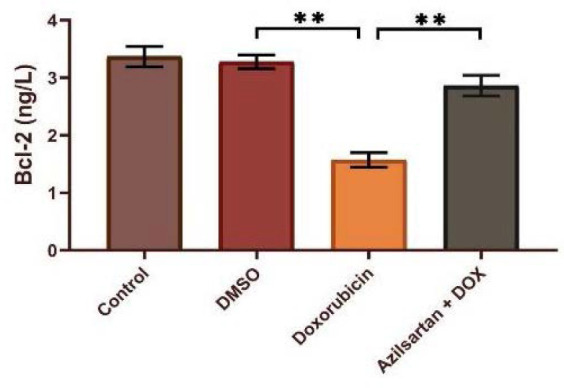
Effect of azilsartan on Bcl-2 levels Rats were administered DMSO (vehicle), DOX (2.5 mg/kg), DOX (2.5 mg/kg) plus azilsartan (5 mg/kg), or were left untreated (control). Data are expressed as mean±SEM. Statistical analysis was performed using one-way ANOVA followed by Bonferroni's multiple comparison test (**p<0.01).

**Figure 7:**
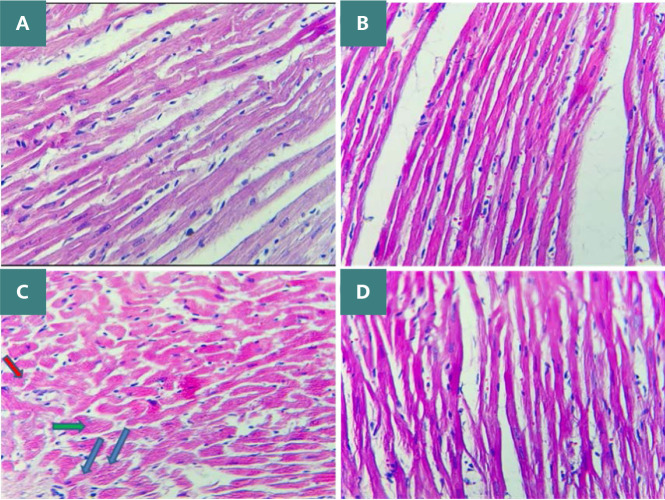
Histopathological assessment of cardiac tissues among the study groups. A: normal histology and no visible damage in the control group. B: DMSO-treated group H&E. C: severe histopathological injury in the myocardium of rats treated with DOX, characterized by disorganized myocardial fibrils, stromal edema, necrosis, pyknotic nuclei (red arrow), perinuclear halo (green arrow), and nuclear fading (blue arrow). D: myocardial tissue of rats treated with DOX and azilsartan showing near-normal histology with organized myocardial fibrils.

Administration of DOX at a dosage of 2.5 mg/kg resulted in significant cardiotoxicity. Compared to the DMSO group, the DOX group had lower levels of TAC and Bcl-2 and increased levels of TNF-α, MDA, IL-1β, and caspase-3. Azilsartan treatment significantly reduced inflammation associated with DOX-induced cardiotoxicity (p<0.05), as evidenced by a decrease in the inflammatory markers TNF-α and IL-1β (Figures 1 and 2). In addition, azilsartan treatment significantly decreased MDA levels in the cardiac tissue of rats (Figure 3) and increased TAC levels (Figure 4). Furthermore, azilsartan significantly reduced cardiac caspase-3 levels, indicating a decrease in DOX-induced apoptotic cell death (Figure 5). Levels of cardiac Bcl-2 were significantly increased in the azilsartan-treated group (Figure 6). Compared to the DOX group, azilsartan significantly improved the scores for the histological lesions associated with cardiomyopathy (Table 1, Figure 7).

**Table 1:** Mean histopathological ascores and comparison between experimental groups

Group	SEM±Mean	Comparison	p-value
Control	0.0±0.0	Control vs. DMSO	0.9747
DMSO	0.0±0.0	DMSO vs. azilsartan	0.2272
Doxorubicin	3.77±0.182	DOX vs. DMSO	<0.0001
Azilsartan	0.35±0.19	DOX vs. azilsartan	<0.0001

## DISCUSSION

DOX, an effective antineoplastic agent, is broadly utilized in the treatment of various cancers, including breast cancer, leukemia, and pediatric malignancies. However, its clinical application is often limited due to a range of systemic adverse effects that accompany its use in tumor chemotherapy. DOX, a DNA topoisomerase II inhibitor, disrupts DNA integrity. Anthracyclines result in dose-dependent cardiac toxicity [6]. Several medications are used to treat DOX-induced cardiotoxicity. Dexrazoxane, for example, when combined with DOX, binds to topoisomerase II and reduces cardiotoxicity and heart failure in patients with delicate cardiac systems. Different techniques, such as beta-blockers, photochemical, and angiotensin inhibitors, demonstrate defense against cardiotoxicity caused by DOX and stop cardiovascular dysfunction. Many studies aimed to identify novel applications of currently available drugs to lessen or prevent DOX cardiotoxicity while maintaining its antitumor-killing capacity [5].

TNF-α is a proinflammatory cytokine with pleiotropy effects that is a member of the TNF-α superfamily. It is well-known for its role in inflammation and its association with various diseases. IL-1β, a key member of the IL-1 family, is essential in the inflammatory response, inducing the production of cytokines, chemokines, and other proinflammatory mediators [16]. In our study, the serum concentrations of TNF-α and IL-1β in the control and DMSO groups showed no significant difference. However, the DOX-treated group had significantly higher cytokine levels than the DMSO group, supporting findings from other research [17-19]. An increase in inflammatory mediators is associated with increased oxidative stress, and these inflammatory responses may be first mediated by NF-κB and result in the transactivation of cytokines with the administration of DOX. Compared to the DOX group, azilsartan significantly decreased the proinflammatory markers IL-1β and TNF-α levels in rats, indicating that it has protective effects against the cardiotoxicity associated with DOX. DOX-induced cardiotoxicity is marked by increased oxidative stress and a continuous generation of oxygen free radicals through various pathways. These ROS can initiate a sequence of lipid-radical reactions, leading to oxidative damage and lipid peroxidation in the mitochondria and cell membranes of myocytes. This lipid peroxidation process gives rise to cytotoxic aldehydes, such as MDA, which can further exacerbate cellular injury [20]. Our study observed an increase in aldehydic lipid peroxide products, including MDA, in the cardiac tissue of rats treated with DOX compared to those receiving DMSO and the control group. The increase in lipid peroxidation was accompanied by a significant decrease in the overall antioxidant capacity of the heart.

These findings align with numerous previous studies indicating that DOX-induced free radicals deplete the antioxidant glutathione and lead to the overutilization of cardiac antioxidant enzymes, including catalase, superoxide dismutase, and glutathione peroxidase. Such oxidative stress could account for the observed reduction in total antioxidant capacity [21-23]. The well-established link between DOX-induced cardiotoxicity and oxidative stress underscores the importance of exploring potential antioxidants. Our research demonstrated that azilsartan preserved the heart tissue's antioxidant status and significantly reduced lipid peroxidation, as indicated by the MDA level reduction and TAC level elevation in rats administered DOX and azilsartan, contrary to rats treated with DOX only. To our knowledge, this was the first study to assess the impact of azilsartan against DOX-induced cardiotoxicity. This study demonstrated a significant increase in caspase-3 activity and decreased Bcl-2 activity within cardiac tissues after DOX administration, compared to the DMSO and control groups. This is consistent with earlier research that showed caspase-3 activation increased after DOX administration in rats [24-26]. Compared to the DOX group, azilsartan prevented the final stages of cell apoptosis, as evidenced by a noticeably decreased level of caspase-3 and an increase in Bcl-2 in cardiac tissue. To our knowledge, this is the first study that evaluated the effect of azilsartan on apoptotic markers—caspase-3 and Bcl-2—in the context of DOX-induced cardiotoxicity. However, a previous study showed a gastroprotective role of irbesartan against ethanol-induced gastric mucosal injury in rats by reducing pro-apoptotic events as evidenced by upregulation of Bcl-2, downregulation of Bax, and inhibition of caspase-3 [27]. Histological analysis of the heart tissue revealed that the myocardium of the DMSO control group maintained normal morphology, characterized by cells of regular size with centrally located nuclei, uniformly arranged with distinct cross-striations, and preserved vascular structure.

Conversely, rats exposed to DOX exhibited significant myocardial changes, including enlarged cell size, cytoplasmic and perinuclear vacuolation, disarrayed myocardial fibers, and loss of myofibrils, as identified in histopathological examinations, supporting prior findings of DOX-induced cardiomyopathy [28-31]. The histopathological assessments in our study revealed a significant improvement in the overall cardiomyopathy scores for the azilsartan-treated group when compared to the DOX-only group, as seen in Table 1, suggesting the ability of azilsartan to attenuate DOX-induced myocardial damage. Azilsartan overlapped with the oxidative pathway, as evidenced by the reduced levels of lipid peroxidation and preserved antioxidant activity in the heart. It also lowered the myocardial inflammatory process, as represented by the reduced concentration of TNF-α and IL-1β, and it prevented the last stage of apoptosis, as demonstrated by the increased Bcl-2 and decreased caspase-3 levels in the tissue of the heart. These findings agree with earlier studies that evaluated the cardioprotective effect of azilsartan in ischemia-reperfusion injury rats. This study showed that pretreatment with azilsartan preserved the cardiac architecture and decreased myofibril loss, necrosis, inflammation, and edema [32]. The lack of cardiac hemodynamic and echocardiographic data represents one limitation of this study.

## CONCLUSION

In conclusion, azilsartan ameliorated DOX-induced cardiotoxicity in rats by interfering with inflammatory responses, apoptosis, and oxidative stress.
